# Transgenic Winter Wheat Expressing the Sucrose Transporter *HvSUT1* from Barley does not Affect Aphid Performance

**DOI:** 10.3390/insects10110388

**Published:** 2019-11-04

**Authors:** Yan Yang, Stefanie Kloos, Isabel Mora-Ramírez, Jörg Romeis, Susanne Brunner, Yunhe Li, Michael Meissle

**Affiliations:** 1 Agroscope, Research Division Agroecology and Environment, Reckenholzstrasse 191, 8046 Zurich, Switzerland; yyhndx@hainanu.edu.cn (Y.Y.); joerg.romeis@agroscope.admin.ch (J.R.); 2 State Key Laboratory for Plant Diseases and Insect Pests, Institute of Plant Protection, Chinese Academy of Agricultural Sciences, 100193 Beijing, China; liyunhe@caas.cn; 3 Key Laboratory of Genetics and Germplasm Innovation of Tropical Special Forest Trees and Ornamental Plants (Hainan University), Ministry of Education, College of Forestry, Hainan University, Haikou 570228, China; 4 Leibniz Institute of Plant Genetics and Crop Plant Research (IPK) Gatersleben, Corrensstrasse 3, 06466 Stadt Seeland, Germany; moram@ipk-gatersleben.de; 5 Agroscope, Research Division Plant Breeding, Reckenholzstrasse 191, 8046 Zurich, Switzerland; susanne.brunner@agroscope.admin.ch

**Keywords:** agricultural biotechnology, cereal aphids, environmental risk assessment, genetically modified (GM) plants, HOSUT wheat, non-target effects

## Abstract

Winter wheat expressing the sucrose transporter *HvSUT1* from barley (HOSUT) has an increased yield potential. Genetic engineering should improve cultivars without increasing susceptibility to biotic stresses or causing negative impacts on ecosystem services. We studied the effects of HOSUT wheat on cereal aphids that feed on the sugar-rich phloem sap. Three HOSUT winter wheat lines, their conventional parental cultivar Certo, and three conventional cultivars were used. Clip cage experiments in the greenhouse showed no differences in life-table parameters of *Rhopalosiphum padi* and *Sitobion avenae* (Hemiptera: Aphididae) on transgenic lines compared to Certo, except higher fecundity of *S.* *avenae* on one HOSUT line. Population development of both aphid species over three weeks on caged flowering tillers did not reveal differences between the HOSUT lines and Certo. When aphids were monitored in a Swiss field study over two years, no differences between HOSUT lines and Certo were observed. We conclude that HOSUT wheat did not have consistent effects on aphids compared to the parental cultivar and measured parameters were generally in the range observed for the conventional winter wheat cultivars. Thus, HOSUT wheat is unlikely to suffer from increased aphid damage.

## 1. Introduction

Genetic engineering (GE) is an important tool for modern agriculture. The GE crops grown today consist mainly of soybean, cotton and maize with tolerance to broad-spectrum herbicides or resistance to Lepidoptera and/or Coleoptera pest herbivores [1]. There is, however, a broad range of other crops and traits under development by the private and the public sectors. Such traits include resistance against pathogens, enhanced nutritional profiles, increased resource efficacy, or tolerance to abiotic stress [2,3].

According to the Food and Agriculture Organization (FAO), wheat is one of the most produced commodities worldwide [4]. Due to its economical and nutritional importance, wheat is the focus of breeding efforts to enhance not only its yield potential, but also to fulfill market demands regarding quality traits like protein content and diverse biotic and abiotic resistance [5].

Several approaches are possible in the quest for enhancing the yield potential of wheat. Among them, grain size and spike fertility are particularly important [6]. Weichert et al. [7] applied a GE approach to improve assimilate partitioning to spikes. Their so-called HOSUT wheat expresses the sucrose transporter *HvSUT1*, controlled by the Hordein B1 promoter, both from barley. HOSUT wheat showed an altered sugar-starch metabolism and assimilate supply to the grains, which translates to increased sucrose uptake capacity compared to the untransformed parental cultivar Certo [7]. Under controlled conditions (pots in the greenhouse with regulated temperature and light) [7,8], under field-like conditions (0.5 m^2^ micro-plots in unregulated 6 × 3 m greenhouses) [9], and under field conditions [7], different lines of HOSUT plants had a superior performance for grain yield, i.e., higher thousand grain weight, more spikes per plant, larger grains, and more endosperm cells in the grains [7,8,9]. In addition, also changes in percentage nitrogen and prolamins, as well as changes in micronutrient content in grains were recorded [7,8,9].

The ectopic expression of the transgenic *HvSUT1* in wheat was designed to be endosperm-specific using the Hordein B1 promoter from barley [10]. In HOSUT line 24, however, expression of the transgene was also found in shoots and roots of seedlings, showing that in this heterologous system, the promoter lost its endosperm specificity [8]. Similarly, expression of the Hordein B1 promoter was reported in vegetative tissues after transformation into rice [11]. In fact, phenotypical differences between HOSUT wheat and Certo, such as increased above-ground biomass, increased number of ears per plant, and decreased total number of tillers per plant, indicate that transgene expression in plant organs other than the grain is likely to affect the plant in the vegetative stage [8]. In addition to direct effects of the expressed transgene on plant physiology, the presence of any transgene and its products, the genomic location of the transgene insertion, or the transformation process itself may lead to unexpected alterations of plant physiology with potential consequences for nutritional quality or ecology [12]. In any case, novel GE plants should not be compromised by increased susceptibility to biotic stress, such as pests and pathogens, or by causing negative impacts on ecosystem functions.

For insect resistance traits, the assessment of potential adverse effects on non-target arthropods has been a major field of public and private sector research [2,13]. For traits other than insect resistance, one way to address potential effects on the food web is to perform experiments with abundant herbivore species that are closely associated with their host plant and are available for testing [14]. A range of arthropod herbivores attack small grain cereals including wheat. Among the most important ones in Central Europe are cereal aphids, e.g., *Rhopalosiphum padi* (Linnaeus), *Sitobion avenae* (Fabricius), and *Metopolophium dirhodum* (Walker)*,* all Hemiptera: Aphididae [15]. Aphids have previously been deployed to study non-target effects of non-insecticidal GE plants, including mildew-resistant wheat [16,17,18], leaf-rust-resistant wheat [19], late-blight-resistant potato [20,21,22], and nematode-resistant potato [23]. For the present study, aphids were selected as test organisms for the following reasons: (1) They are abundant in the field and amenable for testing in the laboratory; (2) Changes in aphid abundance can indicate potential impacts on the food-web, but also on the capability of the plants to cope with biotic stress; (3) Aphids feed on phloem sap and might therefore respond to changes in assimilate and protein supply and expression of regulators related to sugar signaling and amino-acid synthesis [7]. In fact, aphid behavior and performance has been shown to be driven by phloem quality, including sugar, organic acid and amino acid composition in their host plants [24,25,26].

We present experiments under confined greenhouse conditions with *R. padi* and *S. avenae*. Life-table parameters of individual aphids were recorded using clip cages and population development was examined using caged tillers. In addition, aphids were recorded in a two-year open field trial. The main null- hypothesis was that aphid performance is not affected by three HOSUT winter wheat lines compared to the parental cultivar Certo. Potential effects of the HOSUT plants on aphids might be caused by (1) changes in plant physiology as a direct or indirect consequence of the expression of the transgene, i.e., increased assimilate and protein levels or changed expression of genes related to carbon metabolism and amino acid biosynthesis [7]; and (2) unexpected and yet unknown physiological changes in the HOSUT plants caused by the transformation process or the insertion of the transgene.

Three cultivars commercially grown in Switzerland were included in all experiments to illustrate the variability among non-related wheat cultivars. Knowledge of the natural variation among different wheat cultivars helps to interpret whether the magnitude of observed differences among the transgenic lines and their control are likely to be biologically relevant.

## 2. Materials and Methods

### 2.1. Wheat Plants

Three transgenic winter wheat lines, HOSUT 12/44, HOSUT 20/6, and HOSUT 24/31, expressing the sucrose transporter *HvSUT1* from barley under control of the barley Hordein B1 promoter and the corresponding isogenic non-transformed winter wheat cultivar Certo were used. The three HOSUT lines were obtained from three separate *Agrobacterium* transformation events. From the offspring of the transformants, plants homozygous for the HOSUT gene, but without the selectable marker gene, were chosen for further research as described in detail by Saalbach et al. [9]. Using flow cytometry, it was established that each line harbors the transgene in a different part of the genome [27]. The selected HOSUT lines had similar characteristics in yield, and in the morphology, micronutrient content, and protein content of the grain [9]. Certo, the parental cultivar of the HOSUT lines, is classified as a fodder wheat [28]. The transgenic HOSUT lines and Certo were provided by the Leibniz Institute of Plant Genetics and Crop Plant Research (IPK) in Gatersleben, Germany.

Three conventional winter wheat cultivars that are commonly grown in Switzerland, CH Nara, Hanswin, and Sailor (obtained from Delley Samen und Pflanzen AG, Delley, Switzerland), were included in all experiments. For the sake of simplicity, these cultivars will subsequently be referred to as “Swiss cultivars”, although only CH Nara and Hanswin were bred in Switzerland (Agroscope/DSP AG). The three cultivars are very different in yield and protein content [29]. While CH Nara produces top quality grain (high protein content) with comparatively low yield, Sailor produces high quantities of grain in comparatively low quality (low protein content, used as fodder wheat). Hanswin has a medium yield and quality.

All experiments were conducted at Agroscope, Zurich, Switzerland. For life-table and population experiments with aphids, 55 seeds per wheat entry were sown in 5 quick pot trays (51 × 33 cm plastic tray containing 11 × 7 wells) and incubated in the greenhouse. When seedlings were ca. 10 cm high (approximately 10 days after sowing), they were vernalized in a climatic chamber set to 3 °C and a 12:12 h light:dark cycle. After at least 8 weeks, the plants were incubated at 12 °C for ca. 1 week before they were moved to a climate controlled greenhouse chamber at 20 ± 2 °C during the day and 15 ± 2 °C during the night, 70 ± 10% relative humidity (RH), and additional light to ensure a minimum of 16 h light throughout the year. When moved to the greenhouse, part of the plants (those used later for the life-table experiment) were repotted into individual 480 mL plastic pots and the remaining plants (used for the population experiment) were repotted into 3 L pots. Plants in the larger pots were fertilized once with 3 g of slow release fertilizer per pot (Manna, Wilhelm Haug GmbH, Ammerbuch, Germany). Once in the greenhouse, all plants were fertilized weekly with 0.2–0.8 L of 0.2% liquid NPK fertilizer (Manna, Wilhelm Haug GmbH). Plants were positioned randomly to avoid systematic error from varying conditions in the greenhouse chamber. For the experiments with aphids, the wheat plants were transferred to another greenhouse cabin.

### 2.2. Insects

Starter colonies of *R. padi* and *S. avenae* were provided by the Julius Kühn-Institut (JKI) in Quedlinburg, Germany. Subsequently, both aphid species were reared at Agroscope on conventional summer wheat at the 3–4 leaf-stage (Fiorina, top quality grain with high protein content and comparatively low yield [29], UFA Samen, Winterthur, Switzerland) in separate climate cabinets. Rearing conditions were 20 °C, 70% RH during the day (13 h), 15 °C, 80% RH during the night (9 h), and 1 h between the light and dark periods at 17.5 °C, 75% RH. For experiments, aphids were collected from the culturing plants with a fine paintbrush.

### 2.3. Life-Table Experiment (Clip-Cages)

A life-table experiment was conducted in a temperature-controlled greenhouse chamber set to 22 °C and a minimum of 16 h light throughout the year. Plants were used when they had reached the 3–4 leaf stage (ca. 4 weeks after vernalization). Plants in a relatively early vegetative stage were used because: (1) aphid colonization in the field starts in the vegetative stage of the plants; (2) using young plants ensured that green, healthy leaves were present throughout the relatively long duration of the experiment (more than 50 days).

Plants were arranged in a randomized block design, each block consisting of one plant per wheat entry and 10 such blocks were installed for each aphid species (*R. padi* and *S. avenae*). One reproductive adult from the respective culture was caged on the first (oldest) green leaf of each wheat plant using a clip cage. Clip cages (3.5 cm diameter, 1 cm high) had a large hole sealed with fine mesh netting to provide air circulation and foam rubber rings to gently seal them against the leaf [30]. The experiment started with the first offspring in the clip cage (defined as day 0). All aphids except one newly laid nymph were removed from the cage so that the life history of individual aphids could be monitored. Every day, aphid survival and the presence of offspring was recorded. New nymphs were counted and removed. Three repetitions of the described experiment were conducted between March and July 2017, resulting in a total of 30 replications per wheat entry and aphid species. In the first repetition of the experiment, aphids were monitored for 53 days. After this time, most aphids were dead and the ones still alive (one *R. padi* and nine *S. avenae*) had stopped reproducing. When looking closer at the data, we noted that more than 95% of the offspring were produced within the first 35 days. For the second and third repetitions, the experiment was thus terminated on day 36 to ensure that most of the reproductive period was covered and to avoid tedious monitoring of aphids beyond their reproductive period. The nymphal development time was defined as the number of days from day 0 to the day on which first offspring was observed. Total fecundity was the sum of all offspring produced by an aphid during the first 22 days of adulthood. We decided to use 22 days as a cutoff, because this observation period was available for almost all individuals (considering the variation in nymphal development time and the fact that two experimental repetitions were terminated on day 36). To account for early deaths, we also calculated daily fecundity by dividing the 22-day total fecundity by the observed adult period (day of first reproduction to day of death or censoring; for most individuals, this was 22 days).

### 2.4. Population Experiment (Caged Tillers)

A population experiment was conducted in the greenhouse (set to 22 °C, minimum of 16 h light). Plants were used when the first tiller started to flower (ca. 2 months after vernalization). Plants in the early reproductive stage were used for this experiment because: (1) they provide a maximum surface of healthy tissue that can host a large number of aphids; (2) this stage is easy to recognize, which ensures that the experiment starts with plants at the same stage; (3) aphids can choose their position among all possible tissues (leaves, stem, heads).

The Swiss cultivars flowered at least one week earlier than the HOSUT lines and Certo. Therefore, each experiment started with the three Swiss cultivars on one day and with the HOSUT lines and Certo one or two weeks later to ensure that plants were at the same growth stage when starting the experiment. Because of limitations in space and availability of plants, the experiment was repeated multiple times between April 2017 and January 2018. Each repetition consisted of three plants per wheat entry and aphid species. For the HOSUT lines and Certo, four repetitions were conducted, resulting in a total of 12 plants per entry and aphid species. For the Swiss cultivars, six repetitions with a total of 18 plants per entry and aphid species were conducted. The lower number of repetitions for the HOSUT lines and Certo was because of mildew infection in the greenhouse by the end of 2017, which damaged those plants in a way that they could not be used anymore when reaching the flowering stage.

One tiller per plant in the early heading stage was covered with a synthetic gauze bag (50 × 75 cm) that was sealed at the bottom of the tiller with a rubber cord. Access to the bag was possible with a zipper located in the middle of the bag. Five *R. padi* or *S. avenae* adults from the cultures were placed in one clip cage (as described previously) on the top leaf, five aphids on the middle leaf, and five aphids on the bottom leaf of the tiller in the gauze bag. The next day, all adults except five newly laid nymphs per clip cage, and the clip cages themselves were removed. This resulted in 15 neonates per caged tiller that were distributed over three leaves. If fewer than five nymphs were present in any of the clip cages, all nymphs were removed and adults and clip cages remained on the leaves. This procedure was repeated until a sufficient number of offspring was available (day 0). The populations were then left to develop for 21 days. Exceptions were one repetition with *R. padi* and HOSUT/ Certo (3 tillers each), which was terminated after 22 days, and one repetition with both aphid species and the Swiss cultivars (3 tillers each), which was terminated after 20 days because of logistical constraints. For the final evaluation, the caged tillers were cut, the bags were opened cautiously, and all aphids in the bags and on the plants were counted. To facilitate counting, the different parts of each tiller were cut and examined separately. Plant structures were tapped over a large white area with a grid pattern and the fallen aphids as well as the aphids remaining on the plant parts were counted.

### 2.5. Field Experiment

A field experiment with the three HOSUT lines, Certo, and the three Swiss cultivars was conducted in 2017 and 2018 on the protected field site at Agroscope in Zurich, Switzerland [31]. The experiment was arranged in a randomized block design. In 2017, each plot measured 7.5 × 1.5 m, in 2018 7.4 × 1.5 m. The plots within a block were separated by 30 cm walkways, and the blocks were separated by 1 m walkways. Each plot consisted of seven rows of one wheat entry with a density of 350 seeds per m^2^. Each block contained one plot of each wheat entry. The field experiment was surrounded by a 3 m strip of triticale. For the 2017 field season, eight plots per wheat entry were planted on 2 November 2016. For the 2018 season, seven plots per wheat entry were planted on 16 October 2017. Fertilization followed the Swiss guidelines for winter wheat [32]. The selective insecticide spinosad (Audienz, Omya Schweiz AG, Oftringen) was applied against cereal leaf beetles once in 2017 (18 May) and twice in 2018 (14 May and 22 May) at a rate of 0.1 L per ha.

Aphid monitoring was conducted before (17 May 2017 and 14 May 2018) and after the flowering period (13 June 2017 and 11 June 2018). At each sampling date, all aphids were visually counted on at least 24 tillers per plot. The main species *M. dirhodum*, *S. avenae* and *R. padi* were differentiated and individuals that could not be identified were recorded as “unspecified”. Tillers were selected randomly in a way that the sampling covered the whole plot and border rows were avoided. It was also ensured that each member of the evaluation team recorded each wheat entry to avoid systematic bias.

### 2.6. Gene Expression Analysis: RNA Isolation, cDNA Synthesis and qRT-PCR

To confirm the expression of the *HvSUT1* in the HOSUT lines, qRT-PCR was performed on grains 26 days after flowering (DAF). Weichert et al. [7] identified this time point in grain development when the *HvSUT1* transgene has the highest expression in the HOSUT grains. In the second year of the field experiment (2018), seven or eight developing wheat grains were collected from random plants in three plots of each HOSUT line and Certo of the field experiment previously described (three biological replicates). Harvested grains were immediately frozen in liquid nitrogen and kept at −80 °C. Total RNA was extracted using the phenol/chloroform protocol modified from Heim et al. [33]. Briefly, all frozen grains from each plot were ground under freezing conditions. Approximately 50 mg of each ground sample was taken and 700 µL of extraction buffer (1% SDS, 1 M Tris pH 9.0, 10 mM EDTA) and 700 μL phenol/chloroform/isoamyl alcohol (Ph/Ch/I, 25:24:1) were added. The aqueous phase was separated by centrifugation and extracted again with 700 μL Ph/Ch/I. 400 μL of supernatant was taken and the nucleic acids were precipitated over 1 h at −20 °C with 40 μL 3 M Na-acetate and 1 mL absolute EtOH. The resulting pellet was dissolved in 200 μL cold water and RNA was precipitated overnight at 4 °C with 200 μL 4 M LiCl. The RNA pellet was washed twice with 2 M LiCl and cold 70% EtOH. DNase treatment followed with Turbo DNAse kit (Invitrogen, Carlsbad, CA, USA) according to the manufacturer’s instructions.

First-strand cDNA was synthesized with oligo(dT) primers and SuperScript IV reverse transcriptase (Invitrogen, Carlsbad, CA, USA). qRT-PCR was performed using 2X PowerUP Sybr^®^ Green PCR Mastermix (Applied Biosystems, Foster City, CA, USA), with 15–20 ng amplified cDNA in each PCR reaction. For the qRT-PCR three technical replications per plot were analyzed. The used *HvSUT1* (accession no. AJ272309) specific primers were: forward, 5′-CGG GCG GTC GCA GCT CGC GTC TAT T-3′; reverse, 5′-CAT ACA GTG ACT CTG ACC GGC ACA CA-3′. qRT-PCR was performed with ABI Prism 7900HT Sequence Detection System (Applied Biosystems, Foster City, CA, USA), with 2 min at 50 °C, 2 min at 95 °C and 40 cycles with 15 s at 95 °C and 1 min at 60 °C. A dissociation stage of 15 s at 95 °C, 15 s at 60 °C and 15 s at 95 °C followed. Amplification efficiency was calculated with the LinRegPCR program [34]. Expression of the wheat actin gene (accession no. AB181991), measured in all HOSUT lines and Certo, was used for normalization and calculation of the relative expression (E-dCt). Wheat actin-specific primers were used. Forward primer: 5′-GTG GAG GTT CTA CCA TGT TTC CTG-3′; reverse primer: 5′-GCT AAG AGA GGC CAA AAT AGA GCC-3′.

### 2.7. Data Analysis

All the data were analyzed using R, version 3.6.1 (The R Foundation for Statistical Computing, Vienna, Austria). For the life-table experiment, the 22-day fecundity was square-transformed and analyzed using linear mixed effects models (LMER) with wheat entry as fixed factor and experimental repetition as random factor (lme4 package). Daily fecundity was analyzed with untransformed data using the same model and factors. Nymphal development time was analyzed by generalized linear mixed effects models (GLMER) assuming Poisson distribution, with wheat entry as fixed factor and experimental repetition as random factor. In all models, contrasts were set to orthogonal. Comparisons among treatments were analyzed with the Anova function using type III sum of squares (car package). For significant wheat effects, Tukey HSD tests (multcomp package) were used to identify differences between individual wheat entries.

For survival analysis (survival package), a survival object for right censored data was created. Each aphid was either assigned “dead” on the day when the death was observed, or “censored” on the day when the observation period of the individual ended (day 53 for the first experimental repetition, day 36 for the second and third repetitions). Kaplan-Meier estimates were fitted for each treatment. Wheat entries were compared pairwise using the Peto and Peto modification of the Gehan-Wilcoxon test, which weighs the first part of the survival curve higher.

In the population experiment, the Swiss cultivars and HOSUT/Certo did not flower simultaneously, so that the experiment had to be set up with a time shift of one week or more. Given a potential influence of external conditions (e.g., temperature and light) on the climate and consequently population development of aphids in the greenhouse, the Swiss cultivars and HOSUT/Certo were analyzed separately. Data were log-transformed and analyzed by LMER with the fixed factor wheat entry and the random factors experimental repetition, duration of the experiment (usually 21 days, for some repetitions 20 or 22 days), and counting person (variability introduced by different members of the team).

Data of the field experiment were analyzed for all aphid species combined. LMER were used with the fixed factors wheat entry, year and sampling date (first: pre-flowering, second: post-flowering) and the random factor counting person (member of the team recording data for this plot). Because of significant interactions, differences among wheat entries were also analyzed for each year × sampling date combination.

Detectable effect sizes were estimated for the parameters recorded in the greenhouse experiments and the field (pwr package). Those calculations were based on the number of replicates (N), means and standard deviations (SD) of the control cultivar Certo, an alpha-level of 0.05, and a power of 80%. The purpose of those estimates is to get a rough impression how sensitive the different experimental parameters were in each type of experiment with the given number of replicates, which might help when designing future experiments. We did not include the means and SDs of the entries that Certo was compared to, and we did not use power-calculations to interpret statistically non-significant results among different wheat entries [35]. Two-sided *t*-tests were used to estimate detectable effect sizes without taking into account other fixed or random factors. This simplification may have led to estimates of lower power than the statistical models that were actually performed for analyzing treatment differences.

The *HvSUT1* relative gene expression was compared among the three transgenic lines with Anova.

## 3. Results

### 3.1. Life-Table Experiment (Clip-Cages)

The life-table experiment with *R. padi* did not reveal differences among wheat entries for nymphal development time, fecundity in the first 22 days of adulthood, daily fecundity, and survival (Table 1).

Individual *S. avenae* developing in clip cages showed similar nymphal development time among the different wheat entries (Table 1). A weak, but significant, difference among wheat entries was observed for overall survival, but pairwise comparisons revealed no differences for any pair of wheat entries. Fecundity over 22 days and daily fecundity differed among wheat entries. *Sitobion avenae* on CH Nara produced more offspring than on HOSUT 24/31 and Hanswin, and more nymphs were present on HOSUT 12/44 than on Certo, HOSUT 24/31 and Hanswin. The analysis of daily fecundity showed the same effects and in addition, daily fecundity on Sailor was higher than on Hanswin.

Detectable differences with the given means, standard deviations, and Ns of Certo were 18% or lower for all measured parameters (Table 1). Values were generally higher for *S. avenae* (11–18%) compared to *R. padi* (8–13%). The lowest detectable difference was observed for nymphal development time (11 and 8%), the highest for fecundity of *S. avenae* (18%) and survival for *R. padi* (13%).

### 3.2. Population Experiment (Caged Tillers)

**Figure 1:**
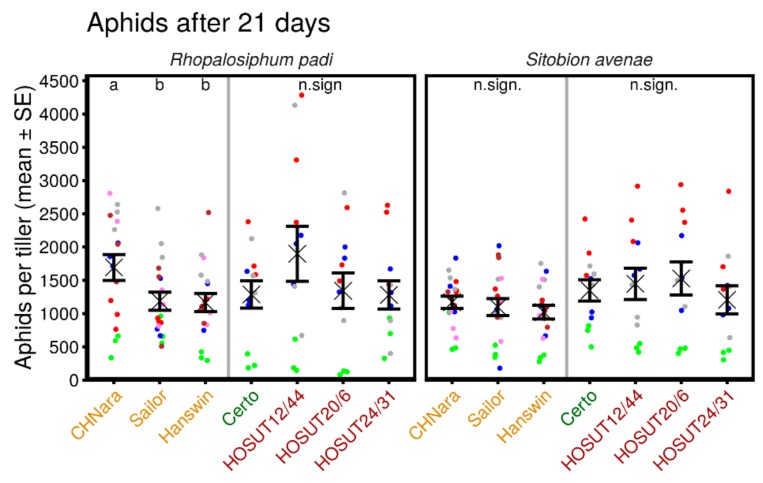
Population size of *Rhopalosiphum padi* (left panel) and *Sitobion avenae* (right panel) caged on one tiller of different winter wheat entries in the greenhouse. The population started with 15 newborn nymphs on each tiller, and all aphids in the cage were counted 21 days later. Statistical analyses were conducted for the conventional Swiss cultivars CH Nara, Sailor, and Hanswin. Separate analyses were conducted for the three HOSUT lines and the non-transformed parental cultivar Certo. Data were analyzed with linear mixed effects models, fixed factor wheat entry, random factors repetition, duration and team member counting the aphids. N = 12 for Certo and HOSUT lines and N = 18 for Swiss cultivars. “n.sign.” indicates non-significant comparisons. Different letters indicate statistical differences (Tukey HSD test). Dots represent data from individual replicates and jittering was applied for better visibility of otherwise overlaying dots. Colors represent experimental repetitions. Please note that the Certo/HOSUT treatments lack two repetitions (brown and purple). Black X-symbols indicate the mean with standard errors.

The populations of *R. padi*, 21 days after infesting individual tillers, did not differ significantly among the three transgenic HOSUT wheat lines and their control Certo (χ^2^ = 3.6, *p* = 0.3) (Figure 1, left panel). In contrast, populations on the Swiss cultivars differed (χ^2^ = 11.7, *p* = 0.003). CH Nara harbored more *R. padi* than Sailor and Hanswin.

No difference was detected for populations of *S. avenae* among the HOSUT/ Certo entries (χ^2^ =5.9, *p* = 0.1) or among the Swiss cultivars (χ^2^ = 2.5, *p* = 0.3) (Figure 1, right panel). The estimated detectable difference based on the means, SDs and Ns of Certo was 66% for *R. padi* and 49% for *S. avenae*.

### 3.3. Field Experiment 

In the first year (2017), a total of 2892 tillers were investigated and 1113 aphids were counted altogether. Most aphids were *M. dirhodum* (754), followed by *R. padi* (222) and *S. avenae* (110). Few aphids (27) could not be classified. In the second year (2018), 2352 tillers were monitored, and the total number of aphids was 1207. The dominating species was again *M. dirhodum* (1091 individuals), followed by *S. avenae* (104) and *R. padi* (12).

The applied statistical model for all species combined demonstrated differences among years and among sampling dates and a strong interaction between years and dates (year: χ^2^ = 62.7; date: χ^2^ = 50.0: interaction: χ^2^ = 111.9; all *p* < 0.0001, interaction plot provided in Figure S1 Supplementary Materials). In 2017, a lower number of aphids were recorded before flowering (0.19 ± 0.03 aphids per tiller, mean ± SE, N = 56 plots) than after flowering (0.60 ± 0.06). In contrast, more aphids were found before flowering in 2018 (0.83 ± 0.09 aphids per tiller, N = 49) than after flowering (0.20 ± 0.04). Approximately half of the aphids observed on the first date in 2017 were winged and consisted mainly of *R. padi*. On the second date, only 5% of the total number were winged aphids. In 2018, few winged aphids were recorded on either date (18 individuals altogether).

**Figure 2:**
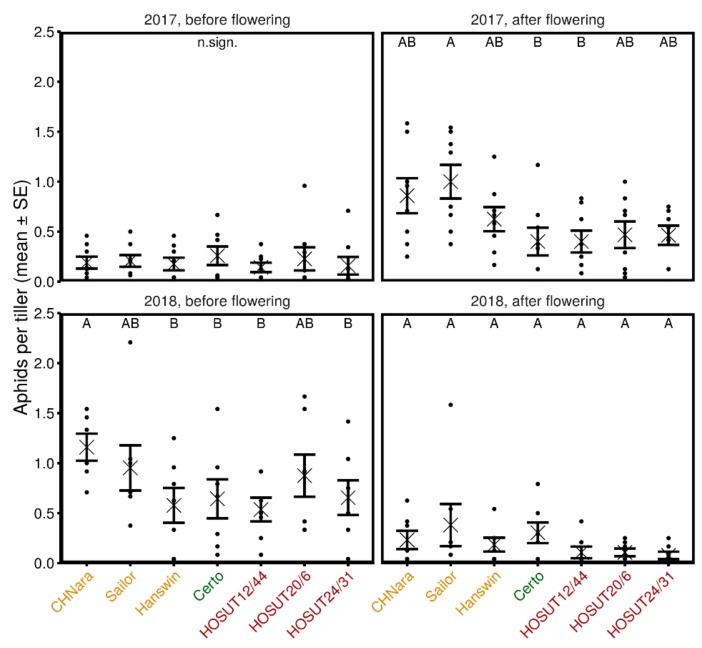
Population size of all aphids recorded on different wheat entries in the field before and after flowering in 2017 and 2018 (Zurich, Switzerland). The field experiment was a randomized block design with 7.5 m (2017) or 7.4 m (2018) × 1.5 m plots and 7 (2018) or 8 (2017) plots (N) per wheat entry. Data were analyzed with linear mixed effects models, fixed factor wheat entry, random factor team member visually counting the aphids. “n.sign.” was used when the model was not significant. Different letters indicate statistical differences (Tukey HSD test). Dots represent data from individual plots, X-symbols indicate the mean with standard errors.

The factor wheat entry was not significant in the overall model (χ^2^ = 1.0, *p* = 0.99), but weak interactions were observed for wheat entry × year (χ^2^ = 14.3, *p* = 0.03) and wheat entry × year × sampling date (χ^2^ = 17.7, *p* = 0.007). Therefore, we analyzed potential differences among wheat entries for each year × sampling date combination separately (Figure 2).

In 2017 before flowering, no statistical difference among wheat entries was present (χ^2^ = 4.4, *p* = 0.7), but after flowering, Sailor harbored more aphids than Certo and HOSUT 12/44 (χ^2^ = 20.0, *p* = 0.003). In 2018 before flowering, more aphids were recorded on CH-Nara than on Hanswin, Certo, HOSUT 12/44 and HOSUT 24/31 (χ^2^ = 17.4, *p* = 0.008). After flowering, the factor wheat entry was borderline significant (χ^2^ = 12.6, *p* = 0.0499), but pairwise comparisons revealed no differences among entries (Figure 2).

Individual aphid species were not analyzed because of many zero-values, in particular for *R. padi* and *S. avenae*. For each species, however, numbers per plot (samples before and after flowering pooled), means and standard errors are visualized in Figure S2 in the Supplementary Materials. Detectable differences estimated for the field experiment, based on the means, SDs, and Ns of Certo, were in 2017 before flowering 151%, after flowering 147%, and in 2018 before flowering 133% and after flowering 147%.

### 3.4. HvSUT1 Gene Expression in Developing Grains

Transgene expression levels were assessed in grains 26 DAF. qRT-PCR of developing grains revealed that expression of *HvSUT1* relative to TaActin 1 in the three transgenic lines ranged between 0.12 and 0.35 (Table S1 Supplementary Materials). Expression-levels in HOSUT 20/6 (0.23 ± 0.046), HOSUT 24/31 (0.26 ± 0.061), and HOSUT 12/44 (0.14 ± 0.011) were not statistically different (Anova, F_2,6_ = 2.0, *p* = 0.2). qRT-PCR also showed the absence of the transgene in Certo grains (relative expression = 0) (Table S1 Supplementary Materials).

## 4. Discussion

The clip cage experiments in the greenhouse showed no significant differences in life-table parameters of *S. avenae* and *R. padi* on any transgenic HOSUT line in comparison with the parental cultivar Certo with one exception. A 15% higher fecundity of *S. avenae* was observed on HOSUT 12/44 than on Certo. The caged tiller experiment revealed no differences in aphid populations between the HOSUT lines and Certo for any of the two evaluated aphid species and the field experiment also showed no effects of the HOSUT lines compared to Certo on aphid abundance. Consequently, the increased fecundity of *S. avenae* observed on HOSUT 12/44 did not translate to a measurable increase in population growth. We cannot exclude, however, the possibility that transformation-related effects in the physiology of HOSUT 12/44 may have influenced the fecundity of *S. avenae* (but not *R. padi*) in the clip cage experiment. In addition, it has to be noted that plants in the vegetative stage were used for the clip cage experiment while flowering plants were used for the caged tiller experiment. Plants in different growth stages may have had different effects on aphids [36,37]. Furthermore, the main aphid species recorded in the field was *M. dirhodum*, while *S. avenae* and *R. padi* occurred in low numbers, which implies that the field data cannot directly be compared to the results of the greenhouse experiments.

Gene expression analysis by qRT-PCR confirmed the expression of *HvSUT1* in the grains of all analyzed HOSUT lines from the field grown in 2018. The three HOSUT lines of this study are homozygous lines for the *HvSUT1* gene and have proven phenotypic stability as well as transgene expression throughout several years of evaluation [8,9]. The plants grown and evaluated in 2018 were descendants of those from 2017; therefore, it is safe to assume that the plants used in 2017 (for the field as well as the greenhouse experiments) also carried a functional form of the transgene.

All our experiments included three wheat cultivars with different properties in protein content and yield that are cultivated in Switzerland [29]. Life-table parameters and abundance of aphids on the three HOSUT lines were generally well within the range of the Swiss cultivars and Certo. In addition, most statistically significant differences in the experiments of this study were observed between a Swiss cultivar and another plant (Table 1, Figure 1 and Figure 2), and not between a HOSUT line and Certo. This confirms that the HOSUT lines did not exhibit uncommon effects on aphid performance exceeding the variability among conventional cultivars.

Several studies have investigated the impact of non-insecticidal GE plants on aphid performance. Effects of non-insecticidal GE plants on aphids were reported for *Myzus persicae* Sulzer (Hemiptera: Aphididae) feeding on potato clones genetically engineered to carry resistance genes against the late blight pathogen *Phytophthora infestans* (Mont.) de Bary [20,21]. Transformation events with different positions of R-gene insertion in the genome influenced the aphids differently [20,21]. Effects, however, were observed only in the first generation, but not in the second generation, and the variability of aphid performance among conventional cultivars was higher than between unmodified and GM potatoes [20]. The same potato lines were also studied by Cascone et al. [22], who found that one event caused increased fertility of *Macrosiphum euphorbiae* (Thomas) (Hemiptera: Aphididae) in the first generation, but not in the second. Von Burg et al. [17] reported that four GE wheat lines carrying a *Pm3b* gene for mildew resistance were of similar host plant quality for 30 different *M. dirhodum* clones compared to the non-transformed control lines. Furthermore, von Burg et al. [18] conclude that effects on the plant–aphid–parasitoid food web between mildew-resistant GE and control lines were inconsistent between study years and in the same range as the variation among wheat cultivars. Similarly, the genetic background of wheat plants had a stronger impact in the performance of *R. padi* than the genetically engineered overexpression of the endogenous *Lr10* gene to enhance resistance to leaf rust [19]. Finally, transgenic potatoes expressing cysteine proteinase inhibitors, which confer resistance to plant-parasitic nematodes, did not affect *M. persicae* compared to the control cultivar [23]. In conclusion, our data and the published literature indicate that GE plants with traits other than insecticidal may or may not affect aphid performance compared to the conventional parental cultivar. Observed effects, however, are often transient or inconsistent, and generally below the magnitude of effects observed among conventional, unrelated cultivars. This confirms the conclusion of more general previous reports that genetic engineering does not lead to unintended effects exceeding those expected from conventional breeding [2,38,39].

The plants that were used in the current study were experimental lines. During the breeding process for the development of new commercial cultivars, lines with unwanted phenotypic or reproductive characteristics (including those caused by transformation-related effects) are excluded from further selection [40]. Minor physiological and metabolic changes that may not be detected phenotypically could remain. Nevertheless, such changes might not be considered biologically relevant [41], in particular when within the variability of the range of commercial cultivars.

Our study covered experiments in the greenhouse with individual aphids, as well as population experiments and a field study. Information on the variability of certain parameters in specific experimental setups can be used to design future studies with sufficient statistical power to be able to detect effects of relevant size if they truly occurred [41]. The value of the greenhouse experiments is a high level of control for seeing potential differences in aphid performance among wheat entries. The estimates of detectable differences, based on the given means, SDs, and Ns of Certo and simple *t*-tests (power analysis), confirm a generally high sensitivity of the measured parameters in the clip cage experiments with values below 20%. More variability was observed in the population experiment with detectable differences of 49–66%. Field studies are valuable for studying plant-environment interactions [42], but the power to detect effects is generally much lower, unless a tremendous sampling effort is undertaken [43]. In our field experiments, the detectable differences were calculated to be up to 150%. Aphids in 2017 and 2018 were present, but not highly abundant with approximately 0.5 individuals per tiller. Similar aphid numbers, however, were reported from a field study with transgenic powdery mildew-resistant wheat conducted in 2008 and 2009 at the same field site in Zurich, Switzerland [16], which indicates that the aphid numbers in the present study were not extraordinary low.

In the field, a range of factors may influence aphid populations, including weather conditions, levels of plant and insect pathogens, and populations of natural enemies. For example, in 2017, aphid populations after flowering were higher than before flowering, while in 2018 the monitoring before flowering revealed more aphids than the one after flowering. The period between the two sampling dates in 2018 was characterized by frequent and heavy rainfall (125 mm in 29 days), in contrast to 2017 (13 mm in 28 days) (nearest weather station REH, Zurich Affoltern). In addition, ca. 3 times more syrphid larvae (which are aphid predators), and pupae were observed after flowering in 2018 than in 2017 (data not shown). Insecticide treatment after the first sampling with spinosad, which has neurotoxic activity in a broad range of insect orders, may also have affected aphid populations. All those factors might have contributed to the population dynamics of aphids in the field. However, our experiment with two sampling dates per year was a snapshot to compare aphid abundances among wheat entries, but not appropriate to explain population curves.

## 5. Conclusions

The presented greenhouse studies provide evidence that the HOSUT winter wheat lines did not systematically affect *R. padi* and *S. avenae*. Furthermore, aphid abundance in the field was similar among the different wheat entries. Previous studies with the HOSUT lines demonstrated that the expression of the transgene resulted in improved yield parameters as well as modified protein and micronutrient levels in the grains. There is also evidence that some differences in the physiology of HOSUT plants compared to the parental cultivar can already occur in the vegetative stage [7,8]. It remains unclear how the genetic modification affects phloem sap quality, including sugar and amino acid composition, which is important for aphid nutrition. In the present study, however, the HOSUT wheat lines did not appear to affect aphid performance compared to the parental cultivar and other conventional wheat cultivars. Thus, HOSUT wheat is unlikely to suffer from increased aphid damage.

## Figures and Tables

**Table 1:** Life-table parameters of *Rhopalosiphum padi* and *Sitobion avenae* reared on 3 transgenic HOSUT winter wheat lines, Certo (non-transformed parental cultivar), or conventional Swiss cultivars (CH Nara, Hanswin, Sailor) in the greenhouse. Values are means and standard errors, N = 30 replicates per wheat entry. Means followed by different letters are significantly different. Detect. diff. indicates an estimate of the statistically detectable difference with α = 0.05 and power = 0.8 based on means, SDs, and Ns of the parental cultivar Certo and simple *t*-tests, regardless of the model that was deployed for the actual analysis.

Aphid Species	Wheat Entry	Nymphal dev. Time (Days) ^1^	Fecundity (# in 22 Days)^2^	Daily Fecundity (# Per Day) ^3^	Survival (Days)^4^
*R. padi*	Certo	7.7 ± 0.15	59.4 ± 1.60	2.7 ± 0.07	42.2 ± 1.32
	HOSUT 12/44	8.3 ± 0.32	58.1 ± 2.10	2.7 ± 0.10	38.8 ± 0.76
	HOSUT 20/6	8.2 ± 0.24	55.8 ± 2.31	2.6 ± 0.09	39.2 ± 1.39
	HOSUT 24/31	8.2 ± 0.22	57.5 ± 2.44	2.8 ± 0.11	37.7 ± 1.65
	CH Nara	7.6 ± 0.15	59.9 ± 2.31	2.8 ± 0.10	37.5 ± 1.26
	Hanswin	7.8 ± 0.14	57.2 ± 1.43	2.6 ± 0.07	42.2 ± 1.24
	Sailor	8.1 ± 0.17	54.9 ± 1.63	2.6 ± 0.09	39.8 ± 1.38
Statistics	Wheat entry	χ^2^ = 1.5, *p* = 0.96	χ^2^ = 5.8, *p* = 0.44	χ^2^ = 8.2, *p* = 0.22	χ^2^ = 11.4, *p* = 0.08
	Detect. diff.	8%	11%	11%	13%
*S. avenae*	Certo	10.2 ± 0.28	46.5 ± 2.10 bc	2.1 ± 0.10 bcd	46.5 ± 1.58
	HOSUT 12/44	9.7 ± 0.22	54.8 ± 2.00 a	2.5 ± 0.09 a	43.7 ± 1.65
	HOSUT 20/6	10.4 ± 0.30	48.7 ± 2.18 abc	2.3 ± 0.09 abcd	48.1 ± 1.56
	HOSUT 24/31	11.0 ± 0.24	43.5± 2.28 c	2.0 ± 0.09 cd	42.8 ± 2.08
	CH Nara	9.8 ± 0.26	53.5 ± 2.68 ab	2.5 ± 0.10 ab	47.6 ± 1.71
	Hanswin	10.3 ± 0.27	42.1 ± 2.14 c	1.9 ± 0.09 d	40.9 ± 1.12
	Sailor	10.2 ± 0.23	49.4 ± 2.43 abc	2.3 ± 0.09 abc	46.9 ± 1.75
Statistics	Wheat entry	χ^2^ = 3.4, *p* = 0.75	χ^2^ = 35.4, *p* < 0.0001	χ^2^ = 40.9, *p* < 0.0001	χ^2^ = 13.3, *p* = 0.04
	Detect. diff.	11%	18%	18%	14%

^1^ Number of days from day 0 to the day when first offspring was observed. Compared with GLMER (Poisson distribution). ^2^ Total number of offspring produced in the first 22 days of adulthood. Compared with LMER after square-transformation. ^3^ Number of offspring produced in the first 22 days of adulthood divided by number of observed days. Compared with LMER. ^4^ Survival based on Kaplan-Meier estimates. Compared with pairwise Peto and Peto tests for wheat entries.

## Supplementary Materials

The following are available online at https://www.mdpi.com/2075-4450/10/11/388/s1, Figure S1: Interaction plot for the mean number of aphids per tiller (all species combined) recorded on different wheat lines in Zurich, Switzerland, Figure S2: Population size of (A) *Metopolophium dirhodum*, (B) *Rhopalosiphum padi*, and (C) *Sitobion avenae* recorded on different wheat entries in the field, Table S1: Calculated relative expression of *HvSUT1* to TaActin 1 (accession no. AB181991.1) analysed in field-collected developing grains 26 days after flowering (2018), Raw data: data used for statistical tests provided as Microsoft Excel spreadsheets.
