# Supplementary material for: Transgenic Winter Wheat Expressing the Sucrose Transporter HvSUT1 from Barley does not Affect Aphid Performance
Source: Insects. 2019 Nov 4;10(11):388. doi: 10.3390/insects10110388 (PMC6920924; doi:10.3390/insects10110388)
Supplement: Supplementary file 1 [file insects-10-00388-s001.zip › ESM1 Supplementary Material FigureS1 S2 and TableS1.pdf]

Supplementary online material

Transgenic winter wheat expressing the sucrose transporter *HvSUT1* from barley does not affect aphid performance

Yan Yang[1,4,5], Stefanie Kloos[1], Isabel Mora-Ramírez[2], Jörg Romeis[1], Susanne Brunner[3], Yunhe Li[4], Michael Meissle[1*]

[1] Agroscope, Research Division Agroecology and Environment, Reckenholzstrasse 191, 8046 Zurich, Switzerland

[2] Leibniz Institute of Plant Genetics and Crop Plant Research (IPK) Gatersleben, Corrensstrasse 3, 06466 Stadt Seeland, Germany.

[3] Agroscope, Research Division Plant Breeding, Reckenholzstrasse 191, 8046 Zurich, Switzerland

[4] State Key Laboratory for Plant Diseases and Insect Pests, Institute of Plant Protection, Chinese Academy of Agricultural Sciences, 100193 Beijing, P. R. China

[5] Key Laboratory of Genetics and Germplasm Innovation of Tropical Special Forest Trees and Ornamental Plants (Hainan University), Ministry of Education, College of Forestry, Hainan University, Haikou 570228, P. R. China.

* Corresponding author:

michael.meissle@agroscope.admin.ch,

Tel. +41 58 468 73 96

Figure S1

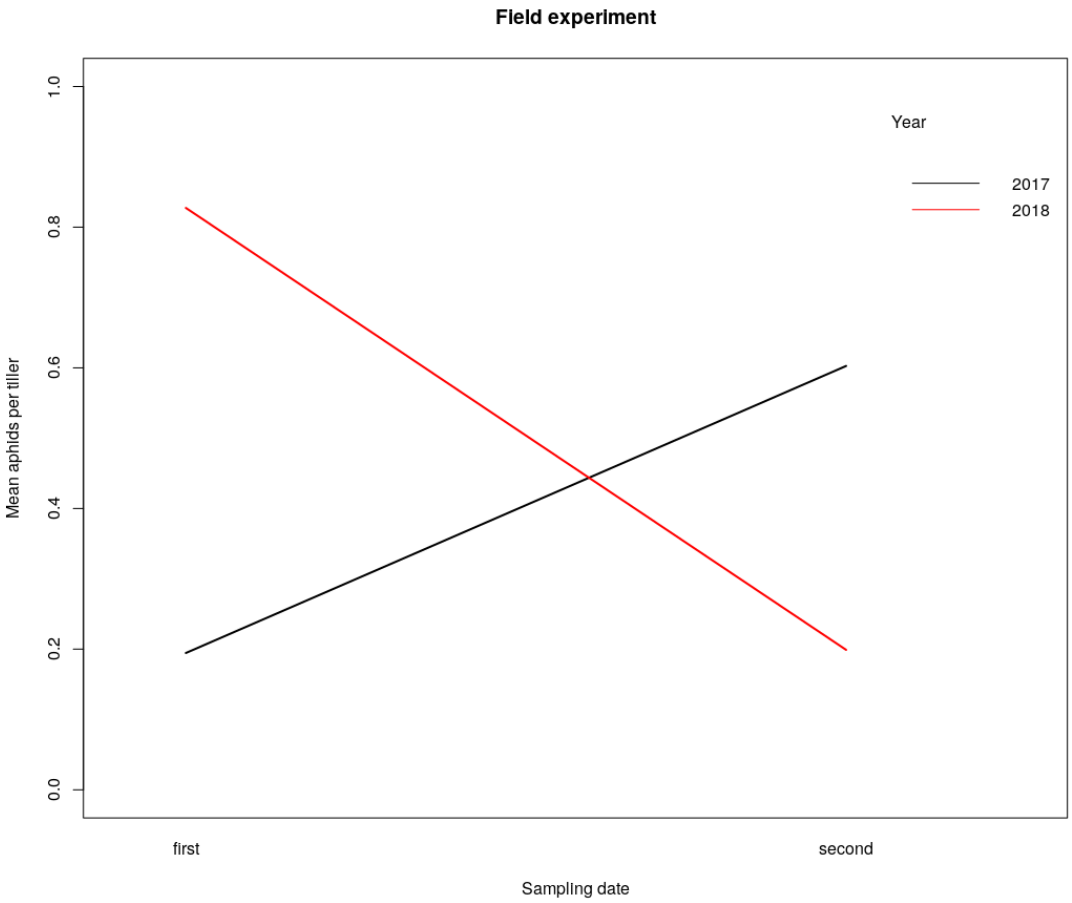

Figure S1. Interaction plot for the mean number of aphids per tiller (all species combined) recorded on different wheat entries in Zurich, Switzerland. The field experiment was a randomized block design with 7.5 m (2017) or 7.4 m (2018) × 1.5 m plots and 8 (2017) or 7 (2018) plots per wheat entry. Each year, one monitoring was conducted before (first sampling date) and one after flowering of the plants (second sampling date). The applied linear mixed effects model (fixed factors wheat entry, year and sampling date, random factor team member counting aphids) indicated strong effects of year, sampling date, and their interaction. Data for 3 genetically engineered HOSUT lines, the parental cultivar Certo, and 3 unrelated, conventional Swiss cultivars were pooled for the purpose of this plot.

Figure S2

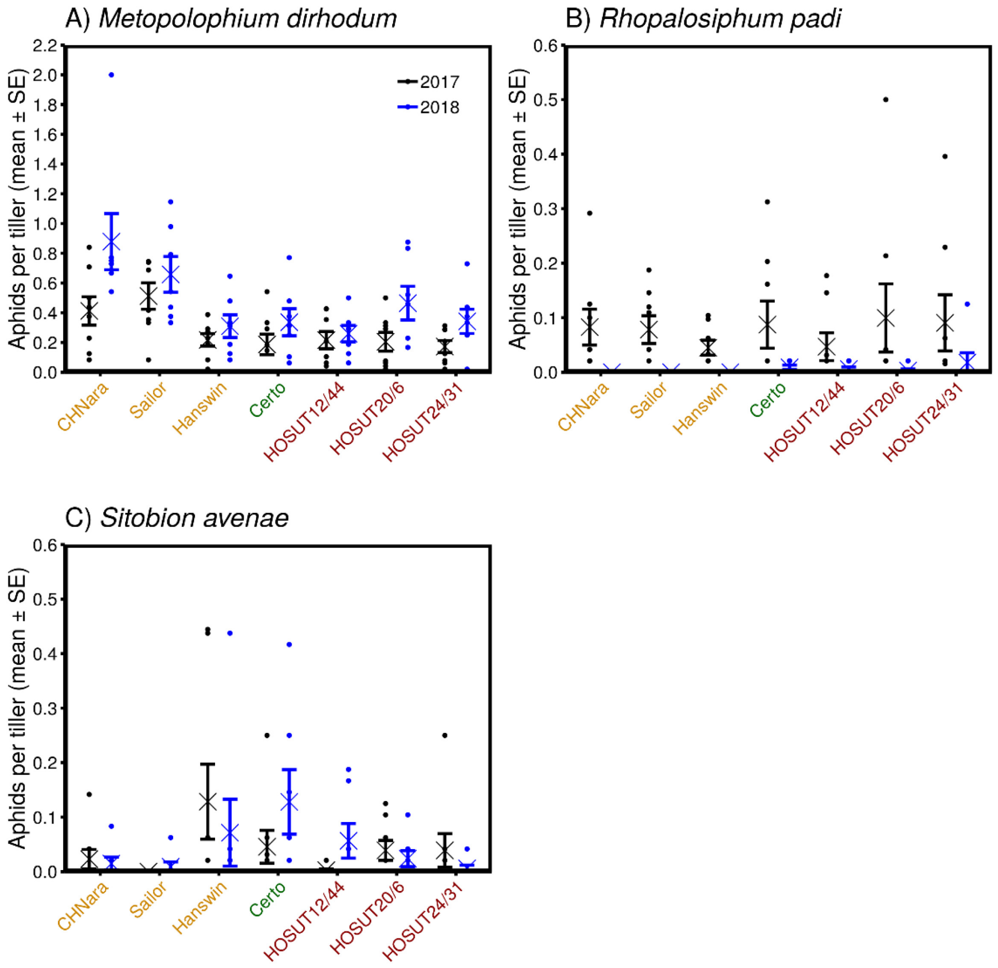

Figure S2. Population size of A) *Metopolophium dirhodum*, B) *Rhopalosiphum padi*, and C) *Sitobion avenae* recorded on different wheat entries in the field. The field experiment was a randomized block design with 7.5 m (2017) or 7.4 m (2018) × 1.5 m plots and 7 (2018) or 8 (2017) plots per wheat entry. Each year, one monitoring was conducted before and one after flowering of the plants. Dots represent individual plots (sampling dates pooled), colours differentiate years. X-symbols indicate the mean with standard errors.

Table S1

Table S1. Calculated relative expression of *HvSUT1* to TaActin 1 (accession no. AB181991.1) analysed in field-collected developing grains 26 days after flowering (2018). Three HOSUT lines and their parental cultivar Certo were analyzed. Means and SEs were calculated over 3 plots (replicates). ANOVA showed no significant differences among HOSUT lines ($p = 0.2$).

Line	Replicate	Rel. Expression (E-dCt)	Mean ± SE
Certo	1	0	
	2	0	0 ± 0
	3	0	
HOSUT12/44	1	0.12	
	2	0.16	0.14 ± 0.011
	3	0.14	
HOSUT20/6	1	0.28	
	2	0.14	0.23 ± 0.046
	3	0.28	
HOSUT24/31	1	0.35	
	2	0.29	0.26 ± 0.061
	3	0.14	
